# Medical History from the Earliest Times

**Published:** 1892-10-15

**Authors:** E. T. Withington


### Medical History from the Earliest Times.

By E. T. Withington, M.A., M.B. Oxon.

### XXVII.—BYZANTINE MEDICINE.

Leo III., the great Isaurian Emperor, had triumphantly defended Constantinople and Christendom against the Saracens, but he failed to preserve his own mind from being affected by the doctrines of the Prophet, whose stern monotheism and horror of images may have seemed a higher form of faith than some aspects of the dominant Christianity. At any rate, in 726 Leo published an edict commanding all pictures and images to be removed from the churches, and thereby inaugurated the iconoclast controversy, lasting more than a century, and ending in the triumph of the orthodox party, which was vigorously supported by monks, by women, and by the Roman pontiffs. The history of the struggle has been written by the victors, who declare that the iconoclast emperors delighted in destroying monasteries and burning libraries, sometimes including the librarians, and that they entirely suppressed all "pious education." Had the result been different, Leo and Constantine might have been handed down to us as champions of Protestantism and progress, but the student of medical history need only remember that the controversy tended to complete the separation of East and West, that many Greeks fled to Italy, some of whom settled at Salerno, and that either from the absence of "pious education," or from some other cause, the history of Byzantine medicine during the whole period is a complete blank.

The Byzantines throughout their entire history appear to have devoted what intellectual energy they possessed mainly to theological controversy. "This city," says St. Gregory of Nyssa, "is full of slaves and mechanics, who are all of them profound theologians. If you want change for a piece of silver, you are informed wherein the Son differs from the Father; if you ask the price of a loaf, you are told in reply that the Son is inferior to the Father; and if you inquire whether the bath is ready, the answer is that the Son was made out of nothing." In the scanty medical writings of the earlier period there is a characteristic tendency to ascribe the origin of medicines to biblical characters and saints, from Ezra, the priest down to St. Gregory the theologian. Here, for instance, is a medical salt invented by the evangelist St. Luke, "which should be used by all pious persons, for it preserves sight, prevents loss of hair, removes phlegm from the chest, favours digestion, fastens loose teeth, &c. Take Cretic hyssop, pennyroyal, thyme, dodder, ginger, salt, ammoniacum, amium, white, black, and long pepper, rub them together into a powder and use like ordinary salt." Myrepsus in the thirteenth century repeats the prescription with slight variations, and declares that the Egyptian hermits consumed great quantities, and were thus enabled to study religious books up to extreme old age.

The tenth century is marked only by the compilation of Theophanes Nonus, which may be fairly characterised as the worst specimen of its class, and consists of carelessly made extracts from the works of older compilers already mentioned. It was written by command of the Emperor Constantine VII., some of whose successors made laudable efforts to encourage science and literature. Among the more noticeable is Alexius I., whose great hospital is described by his learned daughter, Anna, as one of the wonders of the world. But the filial enthusiasm of the princess prevents her giving us any very detailed information. Who shall say, she asks, how many furlongs it is in circumference? Who shall number the thousands of the sick and their attendants it contains? If a stranger visits it in the early morning, it will take him the whole day to go through the wards. The sights seen there resemble those in Solomon's porch, and the dinner hour reminds her of the feeding of the four or the five thousand. Manuel I., grandson of Alexius, was (if we may believe his courtiers) not only the strongest and handsomest man, but also the most skilful physician in his empire. He invented many mixtures, which were long used in the hospitals, and sometimes administered them with his own imperial hands. He could perform the operation of venæsection, and was especially proud of his skill in bandaging, so that when King Baldwin of Jerusalem fell from his horse while hunting and injured his hand, the wound was dressed by the Emperor himself.

Under the patronage of these and other monarchs a partial revival of learning took place in the eleventh and twelfth centuries, marked in medical history by the names of Michael Psellus, Simeon Seth, and Nicetas the surgeon. The first was a man of universal learning, and wrote on every subject from theology to cooking. He introduced the custom of public debates or intellectual tournaments, which he perhaps borrowed from the Arabs, and which afterwards became so prominent a feature of the mediæval universities; but his purely medical works are of little importance, and comprise only a treatise on diet and on the curative virtues of precious stones.

Simeon Seth, as his name indicates, was of Eastern origin, and in his writings the Arabic remedies—manna, nutmeg, musk, cloves, camphor, distilled waters, syrups, juleps, &c.—first become prominent. But he, like Psellus, was primarily a theologian, and the greatest medical work of this period is the "Surgical Collection" made by Nicetas for the use of a Byzantine hospital, and consisting mainly of extracts from the writings of the great operators of the first and third centuries. The Byzantine surgeons, however, probably rather admired than imitated the deeds of their ancestors, and any hopes of further progress were soon extinguished by political disasters.

In the year 1204 a piratical horde, calling itself the fourth crusade, took Constantinople, and the soldiers of the cross having satisfied their fiercer passions, amused themselves by destroying those relics of Greek art and literature which had escaped the flames. For sixty years peace reigned in the Byzantine lecture halls, the peace of desolation, and the first medical work published after the restoration was a sign of relapse rather than of progress. This was the "Dispensarium" or "Dynameron" of Nicholas Myrepsus, a collection of nearly 3,000 recipes from every source, Greek, Arabic, and Salernitan. It includes many universal medicines—panaceas or catholica— as well as prescriptions ascribed to the apostles St. Peter and St. Paul, and the ointment with which St. Mary annointed the feet of Christ, which had since that time been endowed with marvellous curative properties. But perhaps the most interesting is the antidote called Desmoterion, or " the prison," " for we give criminals deadly poison, and then this medicine, and they suffer no hurt. It is also efficacious in removing the pain and stiffness after torture, and in many diseases."

Before saying a final farewell to Greek medicine in its narrower sense, it is pleasant to meet with two physicians, Demetrius Pepagomenus and John Actuarius, whose works are not unworthy of a better age. The former wrote a monograph on gout, and made a praiseworthy attempt to investigate the nature of that disease, which he declares should be treated by diet rather than by drugs, wisely adding that it is easy to give directions on this matter, but hard to carry them out. Actuarius wrote many medical works, the most important being the "De Methodo Medendi," in which he advises that the old drastic purgatives euphorbium, scammony, colocynth, &c., which had been largely replaced by the milder laxatives of the Arabs, should be applied in ointments to the soles of the feet or round the umbilicus, a plan which he had himself employed with great success. Still more interesting is the treatise on the relations of mind and body addressed to his tutor, Rakendytes. That philospher had asked among other things whether it is better for one's intellect to eat once or twice a day. John replies by advising him to take two-thirds of his food at noon, and one-third in the evening, " but if you are unwilling to spend time in doing the same thing twice, or are in the habit of eating only once daily," he may continue to do so, for it will make the mind, if not stronger, at least clearer. While in many respects superior to his predecessors, Actuarius has the same faith in complicated prescriptions, and gives one called "Hygia," whose virtues surpass those of all other panaceas, " for if a man takes a portion of it the size of a bean daily it will not only preserve him from all disease, but also defend him against demons, ghosts, and witchcraft." Like most of the universal medicines it contained opium, but the other ingredients are too numerous to be given here.

